# Identification and Characterization of Sulfated Carbohydrate-Binding Protein from *Lactobacillus reuteri*

**DOI:** 10.1371/journal.pone.0083703

**Published:** 2013-12-31

**Authors:** Keita Nishiyama, Ayaka Ochiai, Daigo Tsubokawa, Kazuhiko Ishihara, Yuji Yamamoto, Takao Mukai

**Affiliations:** 1 Department of Animal Science, Kitasato University School of Veterinary Medicine, Towada, Aomori, Japan; 2 Department of Parasitology, Kitasato University School of Medicine, Sagamihara, Kanagawa, Japan; 3 Graduate School of Medical Sciences, Kitasato University, Sagamihara, Kanagawa, Japan; Charité, Campus Benjamin Franklin, Germany

## Abstract

We previously purified a putative sulfated-galactosylceramide (sulfatide)-binding protein with a molecular weight of 47 kDa from the cell surface of *Lactobacillus reuteri* JCM1081. The aim of this study was to identify the 47-kDa protein, examine its binding to sulfated glycolipids and mucins, and evaluate its role in bacterial adhesion to mucosal surfaces. By cloning and sequencing analysis, the 47-kDa protein was identified as elongation factor-Tu (EF-Tu). Adhesion properties were examined using 6×Histidine-fused EF-Tu (His_6_-EF-Tu). Surface plasmon resonance analysis demonstrated pH-dependent binding of His_6_-EF-Tu to sulfated glycolipids, but not to neutral or sialylated glycolipids, suggesting that a sulfated galactose residue was responsible for EF-Tu binding. Furthermore, His_6_-EF-Tu was found to bind to porcine gastric mucin (PGM) by enzyme-linked immunosorbent assay. Binding was markedly reduced by sulfatase treatment of PGM and in the presence of acidic and desialylated oligosaccharide fractions containing sulfated carbohydrate residues prepared from PGM, demonstrating that sulfated carbohydrate moieties mediated binding. Histochemical staining revealed similar localization of His_6_-EF-Tu and high iron diamine staining in porcine mucosa. These results indicated that EF-Tu bound PGM via sulfated carbohydrate moieties. To characterize the contribution of EF-Tu to the interaction between bacterial cells and PGM, we tested whether anti-EF-Tu antibodies could inhibit the interaction. Binding of *L. reuteri* JCM1081 to PGM was significantly blocked in a concentration-dependent matter, demonstrating the involvement of EF-Tu in bacterial adhesion. In conclusion, the present results demonstrated, for the first time, that EF-Tu bound sulfated carbohydrate moieties of sulfated glycolipids and sulfomucin, thereby promoting adhesion of *L. reuteri* to mucosal surfaces.

## Introduction

Secreted extracellular mucins and cell surface glycocalyx prevent infection by the multitude of microorganisms that live in the healthy gastrointestinal (GI) tract. The secreted mucins that form the mucus layer are produced by specialized mucus-secreting cells, such as paneth or goblet cells, found throughout the GI tract [1]. Underneath the mucus layer, the cells present a dense array of highly diverse mucin glycoproteins and glycolipids forming the glycocalyx [2]. Membrane-anchored cell-surface mucin glycoproteins are a major constituent of the glycocalyx in mucosal tissues. The carbohydrate chains of secreted or cell-surface mucin glycoproteins are highly diverse. Mucin oligosaccharides are joined to the protein core through an initial α-*O*-glycosidic linkage of acetylgalactosamine to the hydroxyl region of serine or threonine. These mucins can be broadly classified into neutral and acidic chemotypes, which are categorized further into sialomucins or sulfomucins on the basis of the presence of terminal sialic acid or sulfate groups, respectively, on the oligosaccharide chains [3], [4].

Lactobacilli are natural inhabitants of the mammalian GI tract and are considered as potential probiotics. Several probiotics enhance GI health by stimulating host immunity and inhibiting pathogen adhesion to the mucosal surface [5]–[7]. One of the desirable properties of probiotics is adhesion to the mucosal surface, which is an important prerequisite for bacterial maintenance in the intestinal tract. Several *Lactobacillus* strains have the ability to adhere to the mucosal surface through the expression of mucins [6], [7], and some strains of intestinal origin display specialized surface adhesins [8]–[10], including mucus adhesion-promoting protein (MAPP) from *Lactobacillus fermentum* 104R [11], elongation factor Tu (EF-Tu) from *L. johnsonii* NCC533 [12], and SpaCBA pili from *L. rhamnosus* GG [13]. Furthermore, mucin-binding protein (MUB) and mucin-binding domain (MucBD)-containing proteins have been reported in several *Lactobacillus* strains [14]–[16]. Although carbohydrate moieties are thought to be responsible for adhesion [14], the detailed structures of binding epitopes of the adhesins on mucin are poorly understood.

We previously reported that *L. reuteri* JCM1081 binds to gangliotetraosylceramide and sulfated-galactosylceramide (sulfatide) [17]. Moreover, *L. reuteri* JCM1081 possesses a cell surface protein that inhibits *Helicobacter pylori* binding to receptor glycolipids, including sulfatide, as demonstrated by thin-layer chromatography-overlay. Cell surface extracts were applied to an agarose gel-immobilized biotinylated galactose (Gal) 3-sulfate probe corresponding to the carbohydrate moiety of the sulfatide, and a protein of approximately 47 kDa was identified as a target candidate sulfatide-binding protein [17]. We further speculated that this protein from *L. reuteri* JCM1081 may bind to sulfomucin and sulfatide because the 3-position of Gal is a common substituent to both moieties. The aim of this study was to identify the 47-kDa protein and evaluate its binding properties to sulfated carbohydrate moieties of glycolipids and mucins. Additionally, we sought to elucidate the role of this protein in bacterial adhesion to the mucosal surface.

## Results

### Identification of a 47-kDa Protein Expressed in *L. reuteri* JCM1081

First, we attempted to identify a cell surface 47-kDa protein, discovered in our previous report as a putative sulfatide-binding protein expressed in *L. reuteri* JCM1081. The N-terminal amino acid sequence of the 47-kDa protein was confirmed by sequencing of the native protein and one of the fragments liberated via limited proteolysis; the AEKEEYE sequence was identical to that described previously [17]. To determine the internal amino acid sequences of the 47-kDa protein, the N-terminal amino acid sequences of the other liberated peptides were determined to be VGLTEDVLKST and EYDFPGDD. When degenerate PCR was performed based on the amino acid sequences, a 499-bp DNA fragment was generated, cloned, and sequenced. The partial amino acid sequence of 166 amino acid residues was deduced from the nucleotide sequence, which shared 100% homology with elongation factor-Tu (EF-Tu) from *L. reuteri* JCM1112 (YP_001841624). To verify the complete sequence of the JCM1081 gene, inverse PCR was performed using a primer set based on the DNA sequence of the fragment generated by the first PCR. The combined nucleotide sequence comprised 1,191 nucleotides with a predicted open reading frame encoding a protein of 396 amino acid residues (molecular mass, 47 kDa). The deduced protein sequence was 100% identical to EF-Tu from *L. reuteri* JCM1112. We concluded that the isolated 47-kDa protein gene from *L. reuteri* JCM1081 was *ef-tu* gene (AB827441).

A BlastP search revealed that the protein sequence of EF-Tu from *L. reuteri* JCM1081 was 59%–73% identical to that of EF-Tu from *Acinetobacter baumannii* (WP_003107886), *Listeria monocytogenes* (NP_466175), *Mycoplasma pneumonia* (WP_010875022), *M. genitalium* (WP_009885583), and *Pseudomonas aeruginosa* (NP_252967). The protein sequences of EF-Tu among *Lactobacillus* species shared around 80%–100% sequence identity. In addition, EF-Tu of *L. reuteri* JCM1081 shared 87% identity with *L. johnsonii* NCC 533 (La1) (NP_964865.1), which is known to bind mucin and epithelial cells [12].

### Binding of His_6_-EF-Tu to Sulfated Glycolipids

**Figure 1:**
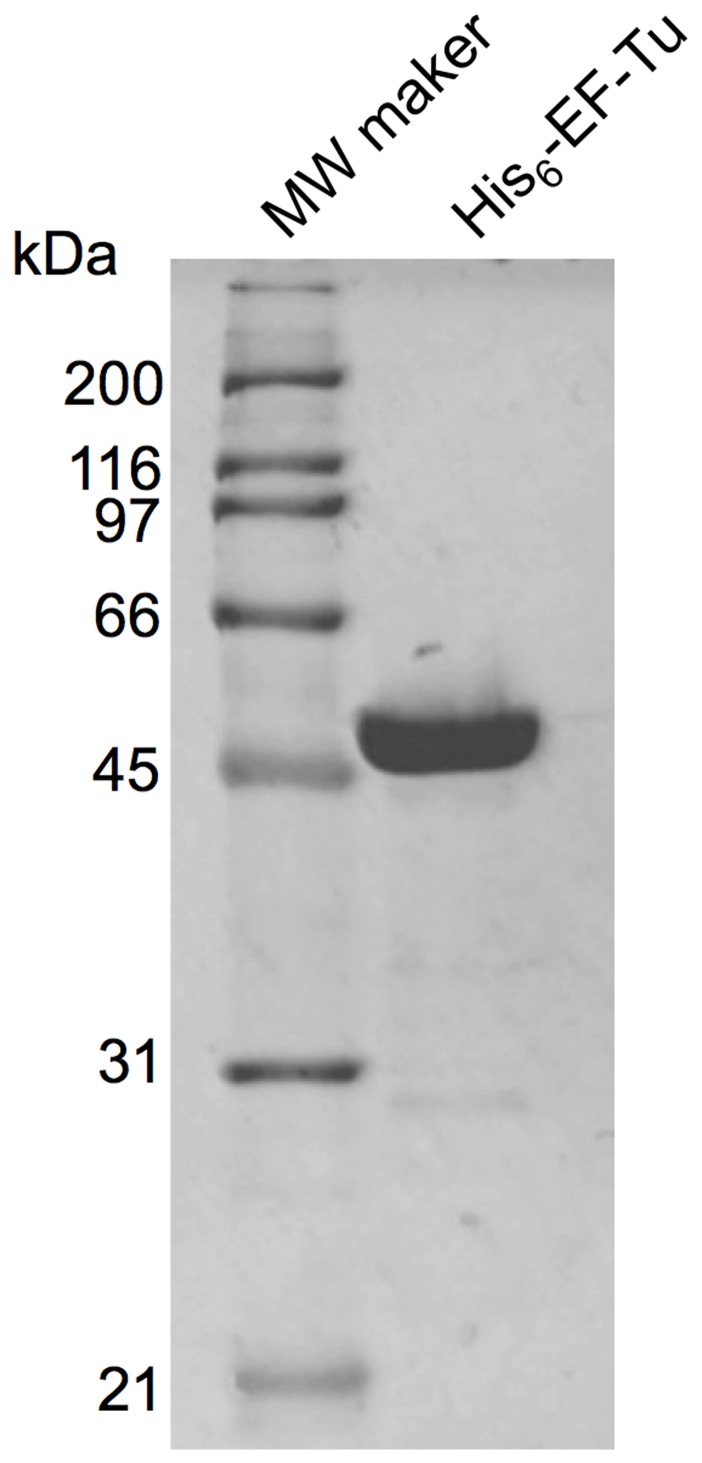
Production and purification of His_6_-EF-Tu. Purified recombinant His_6_-EF-Tu was separated by SDS-PAGE and stained with Coomassie brilliant blue R-250. Molecular mass standards are indicated on the left.

**Figure 2:**
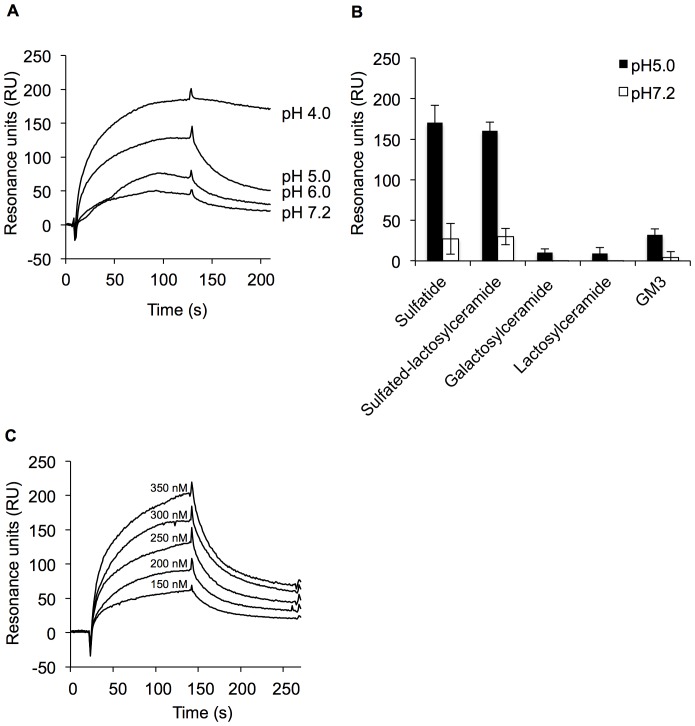
Binding of His_6_-EF-Tu to sulfated glycolipids assessed by SPR analysis. (A) Binding of His_6_-EF-Tu to sulfatide (SO_3_-3Galβ1Cer) at different pH values (pH 4.0, 5.0, 6.0, and 7.2). (B) Binding of His_6_-EF-Tu to various glycolipids: sulfatide, sulfated-lactosylceramide (SO_3_-3Galβ4Glcβ1Cer), galactosylceramide (Galβ1Cer), lactosylceramide (Galβ4Glcβ1Cer), and GM3 (NeuAcα3Galβ4Glcβ1Cer) at pH 5.0 and 7.2. Resonance units were measured at the start of dissociation. Error bars indicate standard deviations (n* = *5). (C) Sensorgrams of the interaction of His_6_-EF-Tu with sulfatide at pH 5.0. Concentrations of His_6_-EF-Tu (from top to bottom) are as follows: 350, 300, 250, 200, and 150 nM. The K_D_ value is described in the text.

6×Histidine-fused EF-Tu (His_6_-EF-Tu) expressed in *E. coli* BL21 was purified on a His-trap HP column, producing a single protein band with a molecular mass of approximately 47 kDa by sodium dodecyl sulfate-polyacrylamide gel electrophoresis (SDS-PAGE; Figure 1), consistent with the calculated molecular mass. We examined binding of His_6_-EF-Tu to sulfatide by surface plasmon resonance (SPR). Since binding of EF-Tu from *L. johnsonii* NCC533 to mucosal surfaces was enhanced at pH 5.0 compared to pH 7.2 [12], binding tests were also performed at different pH values. Lowering the pH from 7.2 to 4.0 increased binding of His_6_-EF-Tu to sulfatide, indicating that His_6_-EF-Tu interacted with sulfatide in a pH-dependent manner (Figure 2A). Next, to confirm binding of His_6_-EF-Tu to the sulfated carbohydrate moieties, binding of His_6_-EF-Tu to several sulfated glycolipids, including sulfatide (SO_3_-3Galβ1Cer) and sulfated-lactosylceramide (SO_3_-3Galβ4Glcβ1Cer), was evaluated by SPR at pH 5.0 and 7.2. Nonsulfated glycolipids, including galactosylceramide (Galβ1Cer) and lactosylceramide (Galβ4Glcβ1Cer), and sialylated glycolipid (GM3; NeuAcα3Galβ4Glcβ1Cer) were also examined. As shown in Figure 2B, His_6_-EF-Tu bound strongly to sulfatide and sulfated-lactosylceramide, and binding was clearly enhanced at pH 5.0. In contrast, there was little binding to galactosylceramide or lactosylceramide at either pH. GM3 binding was only slightly affected by pH, suggesting that sialic acids were not responsible for the interaction. When affinity was evaluated at pH 5.0, concentration-dependent (Figure 2C) between 150 and 350 nM, suggesting that the interaction is specific. The *K*_D_ value for His_6_-EF-Tu binding to sulfatide was estimated to be 5.26×10^−8^ M (*ka*: 6.89×10^2^ M^−1^ s^−1^, *kd*: 3.62×10^−5^ s^−1^). The Chi2 value was calculated to be 3.34; it means that the model used adequately describes our data.

### Binding of His_6_-EF-Tu to Porcine Gastric Mucin (PGM)

**Figure 3:**
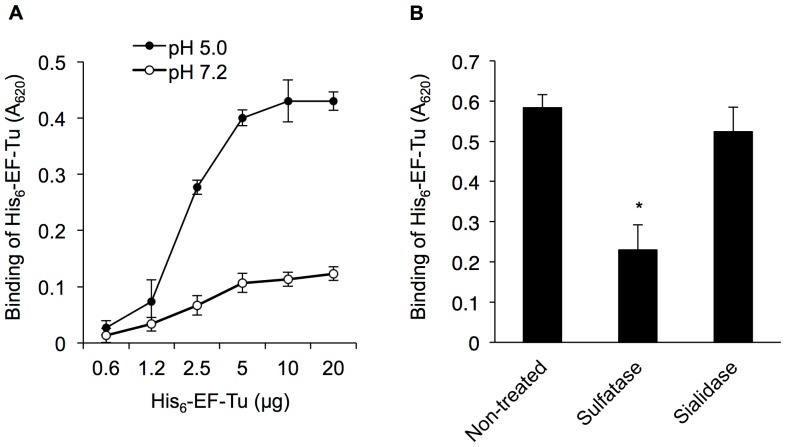
Binding of His_6_-EF-Tu to immobilized PGM. (A) PGM-coated wells were incubated with 0.5–20 µg His_6_-EF-Tu at pH 5.0 or 7.2. (B) Effects of treatment with sulfatase or sialidase on the adhesion of His_6_-EF-Tu to PGM. Detection of bound His_6_-EF-Tu in the presence of anti-His antibodies. Asterisks indicate that His_6_-EF-Tu binding to glycosidase-digested PGM was significantly different (*p<0.05) from binding to untreated control PGM, as determined by Student’s *t*-test. For all studies, error bars indicate standard deviations (n* = *3).

Enzyme-linked immunosorbent assay (ELISA) was performed to determine whether EF-Tu could bind to purified mucin. As shown in Figure 3A, binding of His_6_-EF-Tu to PGM was dose-dependent at pH 5.0 and saturable amounts higher than 5 µg, but had little binding ability to PGM at pH 7.2. We next examined the effects of enzymatic treatment of PGM with sulfatase or sialidase on His_6_-EF-Tu binding. Sulfatase digestion of PGM led to a greater loss of binding than that observed without treatment (*p*<0.05), while sialidase digestion had no effect on binding (Figure 3B), suggesting that EF-Tu bound to PGM via sulfated carbohydrate moieties.

### Sulfated Carbohydrates of PGM were Critical for EF-Tu Binding

**Figure 4:**
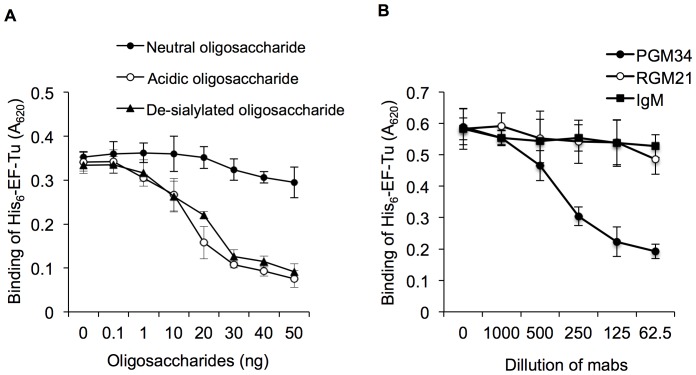
Competition and inhibition of His_6_-EF-Tu-binding to PGM. (A) Binding of His_6_-EF-Tu to PGM was analyzed in the presence or absence of a putative EF-Tu competitor (acidic oligosaccharide and desialylated acidic oligosaccharide or a control neutral oligosaccharide). Each oligosaccharide, at 0.1–50 ng per well as the hexose base, was incubated with His_6_-EF-Tu as described in the Materials and Methods. (B) Inhibition of His_6_-EF-Tu binding to PGM in the presence of monoclonal PGM34 and RGM21 antibodies. Binding tests were performed as described in the text. Mouse IgM was used as a control. Detection of bound His_6_-EF-Tu in the presence of anti-His antibodies. Error bars indicate standard deviations (n* = *5).

We next examined binding of His_6_-EF-Tu to sulfated carbohydrate chains of PGM by competitive enzyme-linked immunosorbent assay (ELISA). Acidic and neutral oligosaccharide fractions of PGM were prepared by alkaline borohydride treatment, gel-filtration chromatography, and anion-exchange chromatography. Desialylated oligosaccharides were prepared from the acidic oligosaccharide fraction by mild acid hydrolysis. Acidic and desialylated oligosaccharide fractions were analyzed by matrix-assisted laser desorption ionization time-of-flight/mass spectrometry (MALDI-TOF/MS). The compositions of all oligosaccharides were assigned to oligosaccharide-alditols, bearing a sulfate or sialic acid residue, as well as *N*-acetylgalactosaminitol at the reducing terminus, based on their masses. MALDI-TOF/MS analysis revealed 23 sulfated oligosaccharide alditols and 8 sialylated oligosaccharide alditols in the acidic oligosaccharide fraction (Figure S1, Table S1). After desialylation by mild acid hydrolysis, the 8 mass ion peaks derived from the sialylated oligosaccharide-alditol spectra disappeared (Figure S1, Table S1). Thus, the composition of acidic and desialylated oligosaccharide fractions was confirmed, and fractions were subjected to binding inhibition tests. As shown in Figure 4A, binding of His_6_-EF-Tu to PGM was inhibited by addition of acidic oligosaccharides in a dose-dependent manner, while the neutral oligosaccharide fraction had little effect on binding. The desialylated acidic oligosaccharide fraction was also tested. Binding inhibition by desialylated acidic oligosaccharide was of the same order of magnitude as inhibition by the acidic oligosaccharide fraction (Figure 4A).

Sulfation of mucin oligosaccharides is primarily linked to the 3-position of the terminal Gal residue, which is also found on the oligosaccharide chain of sulfated glycolipids or the 6-position of *N-*acetylglucosamine (GlcNAc) in the *O*-glycan cores [4]. Thus, we next tested whether the binding of His_6_-EF-Tu to PGM could be inhibited by PGM34 and RGM21 monoclonal antibodies (mAbs), which recognize 6-sulfated blood-group H type 2 and H type 1, respectively [18], [19], as antigens. As shown in Figure 4B, pre-treatment with PGM34 mAb reduced binding of His_6_-EF-Tu to immobilized PGM in a dose-dependent manner, while RGM21 mAb and the IgM control had no apparent effect on binding. Thus, EF-Tu also bound to PGM via the 6-*O*-sulfated GlcNAc residue on PGM.

### Histochemical Study with High Iron Diamine (HID) and His_6_-EF-Tu

**Figure 5:**
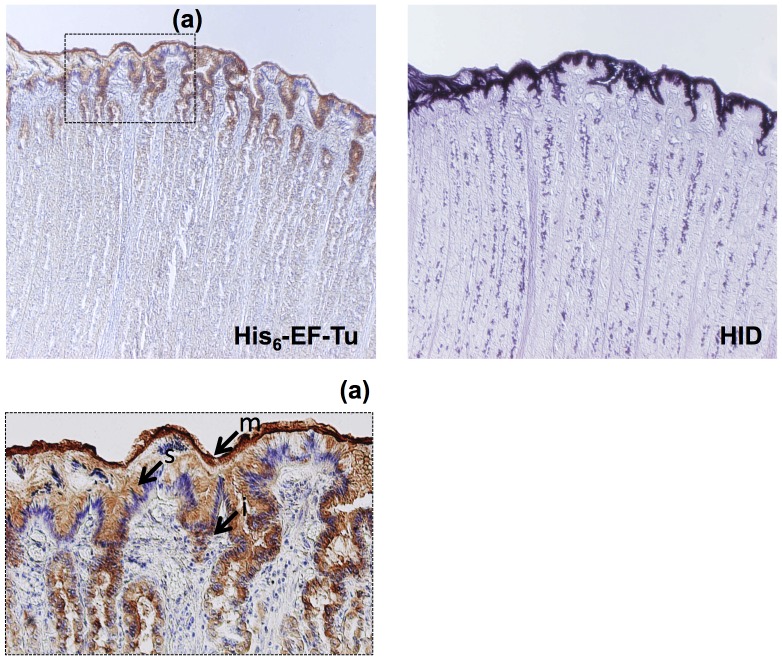
Histochemical staining of the porcine mucosal surface with His_6_-EF-Tu. Binding of His_6_-EF-Tu was observed on the fixed (methanol-Carnoy) mucosal surface (right panels). These areas were coincident with areas positively stained for HID (left panel). Insets (a) are higher magnifications (200×) of the areas indicated by squares in the reference micrographs (40×magnification). Arrows indicate each region: mucous gel layer (m), surface mucous cells (s), and mucous cells around the isthmus region (i).

A histochemical study was performed to determine the reactivity of His_6_-EF-Tu with the porcine gastric mucosal surface. HID staining was used for the detection of sulfated glycoconjugates, including sulfomucin. As shown in Figure 5, the mucous gel layer, surface mucous cells, and mucous cells around the isthmus region were stained with His_6_-EF-Tu (dark brown) in a pattern nearly identical to that of HID, suggesting that EF-Tu interacted with sulfated carbohydrates on the mucosal surface.

### EF-Tu Mediated PGM Adhesion by *L. reuteri* JCM1081

**Figure 6:**
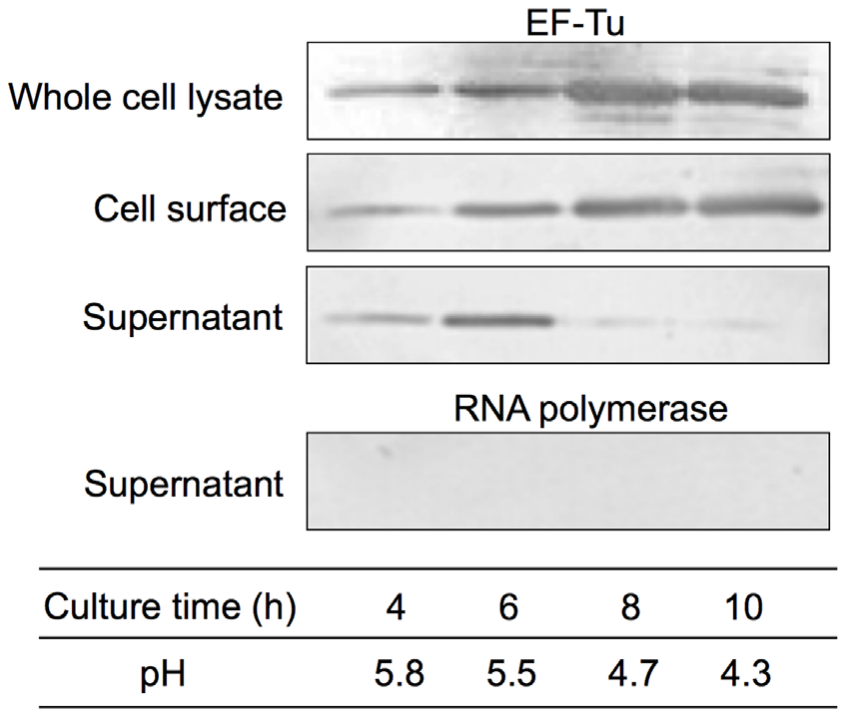
Localization of EF-Tu in *L. reuteri* JCM1081. Supernatant, cell surface, and whole cell lysate fractions at different culture times were analyzed by western blotting. Outer surface proteins of *L. reuteri* JCM1081 were extracted with GHCl. Reactivity with anti-EF-Tu and anti-RNA polymerase antibodies is shown. An anti-RNA polymerase antibody was used to confirm whether cell lysis occurred. Culture time and pH are indicated in the table.

To confirm the association of *L. reuteri* JCM1081 EF-Tu with the cell surface, fractions of cell surface proteins, supernatant, and whole cell lysates were prepared and analyzed by western blotting with anti EF-Tu antibodies. The detected band was abundant in whole cell lysates throughout the culture period, confirming reactivity (Figure 6). EF-Tu in cell surface extracts increased gradually for up to 10 h of culture, indicating the association of EF-Tu with the cell surface. Additionally, EF-Tu was detected in culture supernatants by 6 h and then was dramatically reduced when the pH was raised from 4.7 to 5.5, which is close to the isoelectric point (pI) of *L. reuteri* JCM1081 EF-Tu (pI: 4.9; Figure 6). Moreover, antibodies targeting the cytoplasmic marker RNA polymerase β1 showed no reactivity in the culture supernatant, suggesting that the secretion of EF-Tu was not due to cell lysis.

We next examined whether a shift in the pH mediated the release of EF-Tu into the supernatant. As shown in Figure S2, EF-Tu was detected by western blotting in the supernatant at pH 8.0, but not at pH 4.0. In contrast, EF-Tu was highly expressed in cells incubated at pH 4.0, but only trace amounts were detected at pH 8.0. No trace of RNA polymerase β1 subunit was detected. Therefore, EF-Tu was released into the supernatant at alkaline pH and associated with the cell surface at acidic pH.

**Figure 7:**
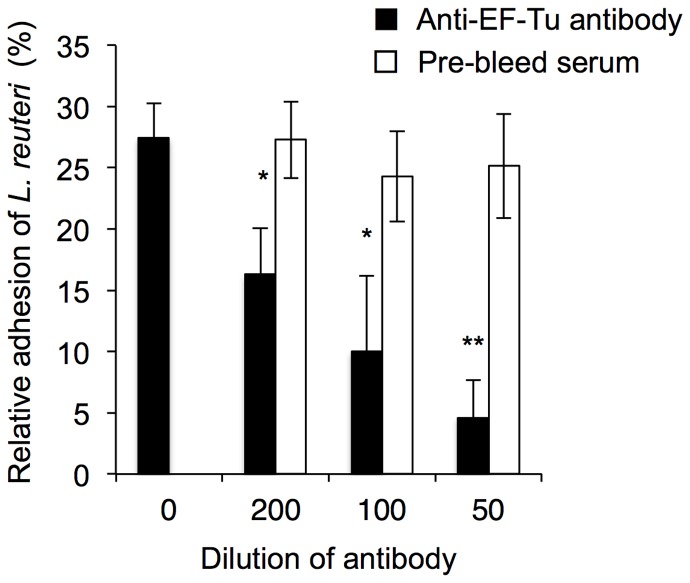
Inhibition of *L. reuteri* JCM1081 binding to PGM using anti-EF-Tu antibodies. Bacteria were pre-incubated with dilutions of anti-EF-Tu antibodies or pre-bleed serum prior to adhesion to the PGM. Adhesion assays were performed as described in the Materials and Methods. Asterisks indicate significant differences in binding (*p<0.05, **p<0.01) compared to antibody-free samples, as analyzed by one-way ANOVA with *post hoc* Dunnet test (n* = *4).

To further characterize the biological role of EF-Tu in the adhesion of *L. reuteri* JCM1081, we examined the effects of anti-EF-Tu antibodies on bacterial adhesion to PGM (Figure 7). We observed a dose-dependent reduction in the binding of *L. reuteri* JCM1081 to PGM, with the maximum inhibition observed at a 1∶50 dilution of the antibody, as compared to the untreated control. Pre-treatment with rabbit pre-bleed serum did not reduce binding. In addition, to verify whether pH could affect bacterial adhesion, adhesion experiments were performed with bacterial cells pretreated at different pH values ranging from 4.0 to 7.2. As shown in Figure S3, bacterial cells pretreated at pH 4.0 showed the highest adhesion capacity to PGM in different pH conditions.

## Discussion

In this study, we demonstrated that the 47-kDa cell surface protein from *L. reuteri* JCM1081, purified in our previous paper as a putative sulfatide-binding protein, could be identified as EF-Tu, is a cytoplasmic protein that interacts with various partners during the elongation cycle of protein biosynthesis. In lactobacilli, the cell surface-associated EF-Tu from *L. johnsonii* NCC533 binds to human intestinal cells and mucins [12]. In addition, upregulation of EF-Tu from *L. plantarum* strains in the presence of mucus suggests that it may play a role in adhesion to the mucosal surface of the GI tract [20], [21]. More recently, EF-Tu has been linked to the adhesion of several *Lactobacillus* species to porcine mucin and reduced enteropathogen adhesion [22]. Although adhesion of EF-Tu to mucosal surfaces has been demonstrated, the mechanism of EF-Tu adhesion remains largely unknown.

Our results showed that His_6_-EF-Tu from *L. reuteri* JCM1081 bound to sulfated glycolipids, but not to nonsulfated glycolipids. Moreover, kinetic analysis showed that the interaction between His_6_-EF-Tu and sulfatide exhibited nanomolar affinity (52 nM) and a slow dissociation rate at pH 5.0. Several reports have demonstrated the binding of *Lactobacillus* strains to carbohydrate moieties of glycoconjugates, some of which were characterized as lectin-like proteins, including glyceraldehyde-3-phosphate dehydrogenase (GAPDH) binding to the human blood group A- and B-antigen [23] and Msa binding to mannose [24], [25]. The affinity of EF-Tu for sulfatide was significantly higher than that of other carbohydrate-binding proteins [23]. Affinities in the nanomolar range (26–89 nM), characterized by slow dissociation kinetics, have been also observed for S-layer protein from *L. brevis* [26]. To the best of our knowledge, this is the first detailed study demonstrating that EF-Tu bound sulfated carbohydrate moieties of glycolipids in a pH-dependent manner.

The carbohydrate chains of mucin glycoproteins are highly diverse and are frequently modified with sulfation of the carbohydrate residues [2]. Sulfation is most often found on the 3-position of Gal and the 6-position of GlcNAc residues of mucin glycoproteins (i.e., sulfomucin) [4]. Sulfation is a critical determinant of the chemical, physical, and biological properties of mucins [27]. Because sulfation confers resistance to most bacterial mucin-degrading enzymes, sulfation has been proposed to provide additional protection for the underlying epithelium against degradation by the high density of resident bacteria [4], [28]. Sulfated carbohydrates are also associated with pathogenic infections, such as *Escherichia coli* [29], *H. pylori* [30]–[34], and *P. aeruginosa* [35]. We found that sulfated carbohydrate moieties also mediated binding of His_6_-EF-Tu to PGM, as demonstrated by ELISA and histochemical experiments. In addition, the results of competitive ELISA using mucin oligosaccharides strongly suggested that neutral and sialic acid-containing carbohydrate moieties as well as core proteins of PGM were not involved in the binding of EF-Tu to PGM. Additionally, our data supported that the sulfated carbohydrate moieties of oligosaccharide chains of PGM were critical for EF-Tu binding, as binding of His_6_-EF-Tu to PGM was inhibited by PGM34 mAb. In addition to revealing the properties of EF-Tu binding to sulfated glycolipids, our results strongly suggested that EF-Tu bound sulfated carbohydrate moieties regardless of the carbohydrate species (i.e., 3-*O*-sulfated Gal or 6-*O*-sulfated GlcNAc residues of PGM). On the other hand, the sulfate residue linked to the 6-position of GlcNAc and the α1,2-linked fucose residue is essential for the reaction with PGM34 mAb [18]. Thus, while EF-Tu has the ability to specifically recognize sulfated carbohydrates of mucin, it may also recognize other carbohydrate structures of PGM34 mAb epitopes.

Interestingly, EF-Tu reacted with sulfated carbohydrates but not sialic acids, although both sulfated carbohydrates and sialic acid residues are negatively charged. Similar interactions have been found in non-fimbrial adhesin coli surface antigen 6 of *E. coli* [29] and soluble neutrophil-activating protein of *H. pylori* [31]; these proteins bind to sulfated glycolipids but not to sialylated glycolipids. In addition, binding of the MucBD-associated domain (MUBAD) from *L. reuteri* to human colonic mucin was reduced by sulfatase digestion but not by sialidase digestion [36]. Whether these binding reactions represent a true recognition process or occur due to complementary ionic interactions remains unknown. One major difference from *L. reuteri* JCM1081 EF-Tu, however, was that binding with *L. reuteri* JCM1081 EF-Tu appeared to depend on pH. Indeed, binding of *L. reuteri* JCM1081 EF-Tu to glycolipids and mucin was enhanced at acidic pH, while little binding occurred at neutral pH. Moreover, SPR interactions between sulfated glycolipid and His_6_-EF-Tu were immediately eliminated by washing with buffer at pH 8.0 (data not shown). We speculated that electrostatic interactions were partially responsible for EF-Tu binding to sulfated carbohydrate moieties. This speculation, however, raises the question of why EF-Tu bound to sulfated carbohydrate residues but not to sialic acid residues. Additional studies are needed to characterize the binding properties of *L. reuteri* JCM1081 EF-Tu to sulfated carbohydrates.

Bacterial cell surface proteins classified as EF-Tu proteins have been identified in *A. baumannii, L. johnsonii, L. monocytogenes, M. pneumoniae*, *M. genitalium*, and *P. aeruginosa* [12], [37]–[42]; however, EF-Tu does not contain known sequence motifs for surface anchoring, nor do the protein sequences contain identified secretory signals. In our study, EF-Tu was confirmed as a secreted protein and was also present on the bacterial cell surface. Interestingly, localization of EF-Tu on the cell surface was associated with pH. Our results are consistent with those described by Antikainen *et al*. [43], who used a stepwise pH increase from 4.4 to 7.0 to find identify the pH at which the release of enolase and GAPDH in *L. crispatus* ST1 becomes detectable in buffer solution at pH 5.2. They also showed that enolase and GAPDH are anchored to the cell surface through ionic interactions and that acidic lipoteichoic acids (LTAs) are the negatively charged anchoring molecules. The pI values of enolase and GAPDH are 4.8 and 5.2, respectively [43]. Taken together, previous reports and our results suggest that EF-Tu is anchored on the cell surface of *L. reuteri* JCM1081 via ionic interactions. In addition, pre-treatment with anti-EF-Tu antibodies dramatically inhibited the adhesion of *L. reuteri* JCM1081 to PGM. Moreover, bacterial adhesion was influenced by pH. Thus, EF-Tu plays an important role in *L. reuteri* JCM1081 adhesion, and an acidic environment is important for the function of EF-Tu as an adhesin on the bacterial cell surface. Thus, the findings of our present study strongly suggested that EF-Tu facilitated the interaction between adherent *L. reuteri* JCM1081 and sulfated carbohydrates, thereby increasing the bacterial capacity of the intestinal environment.

Sulfation is abundant in the carbohydrate moieties of glycoconjugates in human mucosal surfaces; the intestinal mucosa is rich in sulfomucins, with selective secretion of more sulfomucin populations in the rectum [44]. Therefore, interaction with *Lactobacillus* strains and sulfated carbohydrates has received a great deal of attention. *Lactobacillus* ME-522 and *L. gasseri* ME-527 have been recently demonstrated to bind sialic acid and sulfated carbohydrates of human colonic mucin by enzymatic and chemical treatment, although their sulfated carbohydrate adhesin has not been identified [45]. Moreover, the MUBAD found in the surface protein from *L. reuteri* binds to sulfated MUC2 carbohydrate moieties of human colonic mucin and has been proposed as a marker for the diagnosis and prognosis of colonic mucinous carcinoma [36]. However, the involvement of the MUBAD in the adhesion processes of lactobacilli has not been addressed. In this study, we provided the first evidence that EF-Tu bound to sulfated carbohydrate moieties of glycoconjugates, including mucins and sulfated glycolipids, and participated in the adhesion of *L. reuteri* JCM1081 to mucosal surfaces. Moreover, we identified pH-dependent binding properties of *L. reuteri* JCM1081 EF-Tu, which were similar to those described by Granato *et al*. [12], who found that the recombinant EF-Tu protein of *L. johnsonii* NCC533 bound efficiently to mucins at pH 5.0, but not at pH 7.2. The amino acid sequence of EF-Tu shared high identity with those of *L. johnsonii* NCC533 and other *Lactobacillus* strains. Thus, we hypothesize that binding of EF-Tu to the mucosal surface is mediated by sulfated carbohydrates in other *Lactobacillus* species as well. Further studies will be required to identify the properties of EF-Tu binding to sulfated carbohydrates in some *Lactobacillus* species.

## Materials and Methods

### Ethics Statement

No ethical approval was required for tissue collection because the tissue was obtained from a local slaughterhouse (Towada, Aomori, Japan), and the animals were not killed for scientific research. Permission was obtained from the slaughterhouse to use the collected animal parts.

### Bacterial Strains and Culture Conditions

*L. reuteri* JCM1081 was obtained from the Japan Collection of Microorganisms and cultured on De Man-Rogosa-Sharp (MRS) agar plates (BD Difco, Le Pont de Claix, France) at 37°C under anaerobic conditions. *E. coli* strains DH5α and BL21 (DE3) (Stratagene, La Jolla, CA, USA) were grown in Luria-Bertani (LB) broth or on LB agar plates at 37°C. Ampicillin (100 µg/mL) and kanamycin (50 µg/mL) were added when necessary.

### Amino Acid Sequence Analysis

Extraction of bacterial surface proteins was performed with octyl-β-d-glucopyranoside (Wako, Tokyo, Japan) as described previously [17], and the 47-kDa protein (EF-Tu) was purified using SDS-PAGE. To analyze the N-terminal and internal amino acid sequences of EF-Tu, we performed limited proteolysis with V8 protease. The protease digests were separated by tricine SDS-PAGE as described by Ploug *et al*. [46] and electroblotted to an Immobilon-P^SQ^ membrane (Millipore, Bedford, MA, USA). The targeted bands were excised and analyzed on an Applied Biosystems Procise 491 protein sequencer.

### Cloning and DNA Sequencing

A portion of the *ef-tu* gene was amplified by degenerate PCR using a primer set designed for the N-terminal and internal amino acid sequences of EF-Tu as follows: 5′-GGIGA(A/G)AA(A/G)GA(A/G)CA(C/T)TA-3′ and 5′-TCICCIGG(A/G)AA(A/G)TC(A/G)TA(C/T)TC-3′. The amplified fragment was cloned and sequenced. Next, inverse PCR was performed with the primers 5′-ACTGGTGCTGCACAAATGGATGG-3′ and 5′-GATCATGTTCTTAACGTAGTCAGCGT-3′; the amplified fragment was cloned and sequenced. The complete DNA sequence of the *ef-tu* gene was determined by sequencing these amplicons using a BigDye Terminator v3.1 Cycle Sequencing Kit (Applied Biosystems, Foster City, CA, USA). Electrophoresis was performed on an ABI PRISM 3100 Genetic Analyzer (Applied Biosystems). Protein sequence homology searches were performed with BlastP (http://blast.ncbi.nlm.nih.gov/Blast.cgi).

### Expression and Purification of Recombinant Protein

The expression vector pET28b (Novagen, Madison, WI, USA) was engineered to express the recombinant *ef-tu* gene with a 6×His tag fused to the N-terminus. The *ef-tu* gene was amplified with the primers 5′-CATATGGCTGAAAAAGAACATTATGAAC-3′ and 5′-CTCGAGTTAGTCTAAGATGTCGGATAC-3′, introducing *Nde*I and *Xho*I sites (underlined). The amplicon was digested with *Nde*I and *Xho*I and ligated into pET28b. The resulting plasmid was confirmed by sequencing and introduced into *E. coli* strain BL21. Transformed cells were grown in LB medium at 37°C with shaking. When the OD_600_ reached 0.5, isopropyl-β-d-thiogalactopyranoside (IPTG, 0.5 mM) was added to induce protein expression. After cultivation at the same temperature for 5 h, the cells were harvested and lysed in BugBuster protein extraction reagent (Novagen) to obtain a cell-free extracts. His_6_-EF-Tu was purified by Ni^2+^-nitrilotriacetic acid affinity chromatography (GE Healthcare, Piscataway, NJ, USA). Protein purity was assessed by SDS-PAGE (12.5% polyacrylamide), and concentrations were determined spectrophotometrically by the BCA method (Thermo Scientific, Wilmington, DE, USA). Antisera against purified His_6_-EF-Tu were raised in rabbits by routine immunization procedures.

### Purification of PGM

PGM was purified by gel filtration chromatography and cesium chloride (CsCl) density-gradient ultracentrifugation as described previously [47]. Briefly, powdered porcine gastric mucin (Wako) was suspended in 2% TritonX-100, 50 mM Tris-HCl buffer (pH 7.4). The suspension was centrifuged at 10,000×*g* for 30 min at 4°C and then applied to Sepharose CL-6B column (GE-Healthcare, 2.5×100 cm). The void volume fractions were corrected, dialyzed against distilled water, and lyophilized. CsCl (Sigma-Aldrich, St. Louis, MO, USA) was added to the solution of lyophilized crude mucin to adjust its mean specific density of ∼1.4 g/mL. The resulting solution was ultracentrifuged at 150,000×*g* for 90 h at 20°C. Hexose peaks of the fraction with a specific density of ∼1.4 g/mL were collected and dialyzed against distilled water. Hexose was determined by the phenol sulfuric acid method. Protein concentration was measured using a BCA Protein Assay kit (Thermo Scientific).

### Enzymatic Treatment of PGM

Lyophilized PGM (10 mg) was treated with 4 mU of *Clostridium perfringens* sialidase (Roche, Indianapolis, IN, USA) in 50 mM acetate buffer, pH 5.0, or 6 mU of *Aerobacter aerogenes* sulfatase (Sigma-Aldrich) in 50 mM Tris-HCl pH 7.1 containing 100 mM KCl and 10 mM MgCl_2_. Reaction mixtures were incubated for 4 h at 37°C. After incubation, the digested mucins were separated by gel filtration on a Sepharose CL-6B column.

### Preparation of Oligosaccharide Fractions from PGM

Porcine mucin oligosaccharides were prepared and identified according to the method described by Tsubokawa *et al*. [18]. Briefly, oligosaccharides released from PGM by alkaline borohydride treatment were fractionated by gel filtration. The acidic oligosaccharide fractions were purified by anion-exchange chromatography. To prepare the desialylated oligosaccharide fraction, acidic oligosaccharide fractions were hydrolyzed with 0.1 M HCl at 80°C for 1 h. Sulfated carbohydrate residues were then fractionated using a Bio-Gel P10 column (Bio-Rad Laboratories, Hercules, CA, USA). The molecular masses of the desialylated acidic oligosaccharides were determined by MALDI-TOF/MS using a Voyager DE-PRO instrument (Applied Biosystems) in negative ion mode.

### SPR

The binding affinity of EF-Tu for glycolipids was assessed by SPR on a Biacore X instrument (GE Healthcare). His_6_-EF-Tu was covalently immobilized on CM5 dextran sensor chips using amine-coupling chemistry reagents (GE Healthcare). Approximately 2,500 resonance units (RU) of His_6_-EF-Tu were immobilized. All experiments were performed at 20 µL/min using ABS-EP (10 mM acetate buffer containing 150 mM NaCl, 3.4 mM EDTA, 0.005% surfactant P20; pH 4.0, 5.0, or 6.0) or HBS-EP (10 mM HEPES containing 150 mM NaCl, 3.4 mM EDTA, 0.005% surfactant P20; pH7.2) at 25°C. The following glycolipids were used as analytes: sulfatide, sulfated-lactosylceramide, galactosylceramide, lactosylceramide, and GM3. The analytes were diluted in buffer and injected at 350 nM for the binding assay. RU values were measured at the start of dissociation without further sample addition. The signal from each binding experiment was corrected for nonspecific binding by subtracting the signal obtained from the blank surface. Finally, regeneration of the sensor surface was achieved with a 60-s exposure to 50 mM HCl (pH 8.0). For the kinetic assay, 150−350 nM sulfatide was injected over immobilized His_6_-EF-Tu at pH 5.0. The dissociation step was performed at the same flow rate of 120 s. The association rate (*ka*), dissociation rate (*kd*), and dissociation constant (K_D_ = *kd*/*ka*) were calculated using BIA evaluation version 3.0 (GE Healthcare). Global analysis was performed using the simple 1∶1 Langmuir binding model. Goodness of fit was indicated by Chi2, and values of less than 10 indicated good fit.

### ELISA

A 96-well microplate was coated with 100 ng purified PGM as the hexose equivalent followed by blocking with phosphate-buffered saline (PBS) containing 2% (w/v) bovine serum albumin (BSA) for 2 h at room temperature. After washing the PGM-coated wells twice with ABS (10 mM acetate buffer containing 150 mM NaCl; pH 5.0) or HBS (10 mM HEPES containing 150 mM NaCl; pH 7.2) containing 0.1% (w/v) BSA, His_6_-EF-Tu was added and incubated for 1 h. After washing 3 times, anti His-tag mouse IgG (Roche; 1∶1500 dilution) was added and incubated for 1 h at room temperature. Alkaline phosphatase (AP)-conjugated anti-mouse IgG (Dako, Glostrup, Denmark;1∶2000 dilution) was added and incubated for 1 h at room temperature. Wells were developed with a BluePhos MicroWell Substrate (KPL, Gaithersburg, Maryland, USA).

### Competitive ELISA

Competitive ELISA was performed to detect the interaction between His_6_-EF-Tu and PGM oligosaccharides. Each oligosaccharide fraction (0.1−50 ng as a hexose equivalent) was pre-incubated with 10 µg His_6_-EF-Tu for 1 h at room temperature. The mixtures were added to the PGM-coated wells and incubated for 1 h. After washing 3 times, anti His-tag antibody (1∶1500 dilution) was added and incubated for 1 h at room temperature. Subsequent steps were performed as described above.

For the inhibition assay, PGM34 mAb and RGM21 mAb (Kanto Chemical, Tokyo, Japan; 1∶62.5−1∶1000 dilutions) were added to the PGM-coated wells, followed by incubation for 1 h at room temperature. Mouse IgM was used as a control. After washing twice with 0.1% (w/v) BSA-ABS (pH 5.0), His_6_-EF-Tu (10 µg) was added to each well and incubated for 1 h. After washing, biotinylated anti His-tag mouse IgG (1∶1000 dilution) was added and incubated for 45 min. The wells were then washed and incubated with AP-conjugated avidin (1∶2000 dilution; Roche) for 30 min at room temperature. Subsequent steps were performed as described above.

### Histochemistry

Gastric tissue of 24-week-old conventional swine was obtained from a local slaughterhouse immediately after death. Gastric tissue samples were fixed without washing in methanol-Carnoy’s fixative for 4 h at 37°C. Paraffin-embedded sections were dewaxed, hydrated, and cut into 4-µm sections. Histochemical detection of whole sulfated glycoconjugates was performed by HID staining [48]. The paraffin sections were treated with diamine solution (containing N,N-dimethyl-m-phenylenediamine, N,N-dimethyl- p-phenylenediamine, and iron chloride) for 20 h at room temperature. The sections were briefly washed with distilled water, dehydrated, passed through xylene, and mounted. Pictures were obtained with an Olympus BX53 microscope (Tokyo, Japan).

For the binding test, endogenous peroxidase activity in the sections was blocked, and sections were incubated with 5% (w/v) BSA-PBS for 1 h. Biotinylated His_6_-EF-Tu (30 µg) was suspended in 1% (w/v) BSA-ABS (pH 5.0), and the mixture was incubated for an additional 12 h at 4°C. After washing, peroxidase-conjugated streptavidin (Roche) (1∶1000) was added and incubated for 60 min. Sections were then immersed in color-developing reagent using ImmPACT DAB Substrate (Vector Laboratories, Burlingame, CA, USA). Counterstaining was performed with hematoxylin.

### Preparation of Cell-surface Proteins

*L. reuteri* JCM1081 was cultivated anaerobically in 100 mL MRS broth at 37°C for 4−10 h. Cell-surface protein was extracted as described by Lortal *et al.* [49]. Briefly, bacteria (10^10^ cells) were harvested by centrifugation (16,000×*g*, 10 min, 4°C) and incubated with 5 M guanidine hydrochloride (GHCl) for 15 min at 4°C, followed by an additional centrifugation at 16,000×*g* for 10 min at 4°C). The extract was dialyzed against 25 mM Tris-HCl (pH 7.0) containing 1 mM EDTA at 4°C for 48 h. Culture supernatants were filtered through syringe filters with a 0.2-µm pore size. Whole cell lysates were resuspended in an equal volume of 25 mM Tris-HCl (pH 7.0) containing 1 mM EDTA and 0.3 g 0.1-mm zirconia-silica beads. Total suspension was achieved by beating for 180 s at 4,800 rpm in a bead beater (FastPrep QBiogene, Carlsbad, CA, USA). The debris was removed by centrifugation at 10,000×*g* for 5 min at 4°C; the supernatants were collected for western blotting.

To study EF-Tu detachment from the cell surface, bacteria (10^10^ cells) were harvested by centrifugation. Bacterial pellets were suspended in ABS (pH 4.0) or HBS (pH 8.0) and incubated at 37°C for 1 h; the cells and supernatant were separated as described above.

### Western Blotting

Protein samples were separated by SDS-PAGE (12.5% polyacrylamide) and transferred to polyvinylidene difluoride membranes. Membranes were blocked with 5% (w/v) skim milk in PBS-0.05% Tween 20 (PBS-T) for 2 h at room temperature. After washing membranes with PBS-T, anti EF-Tu antibodies (diluted 1∶1500 in PBS-T) were added. Membranes were then washed and incubated with AP-conjugated mouse anti rabbit IgG (Dako) diluted 1∶2000 in PBS-T at room temperature for 1 h. After washing, the signals were developed with a BCIP/NBT liquid substrate system (Sigma-Aldrich). Anti-RNA polymerase antibodies (1∶2500 dilution; Acris Antibodies, San Diego, CA, USA) were used to verify that cell lysis had occurred.

### Bacterial Adhesion to PGM

A 96-well microplate was coated with 100 ng PGM (100 µL/well) followed by blocking with 2% (w/v) BSA-PBS for 2 h at room temperature. *L. reuteri* JCM1081 was cultivated in 5 mL MRS broth at 37°C for 10 h. Cells were harvested by centrifugation (6,000×*g*, 5 min, 4°C). For pH treatment, bacteria (5×10^7^ colony-forming units [CFU]/mL) were suspended in DMEM (pH 4.0, 5.0, 6.0, or 7.2) and incubated for 1 h at 37°C. Bacterial suspensions were added to each well, and plates were incubated for 1 h at 37°C. After washing twice, 100 µL of 0.1% Triton X-100 in PBS was added to each well, and bacterial cells were suspended by vigorous pipetting. Serial dilutions of suspended bacteria were plated on MRS agar. Adhesion to PGM was expressed as a percentage calculated from 4 independent experiments, as follows: 100×(number of adhesive bacteria/number of bacteria inoculated). To determine whether antibodies against EF-Tu reduced *L. reuteri* JCM1081 binding to PGM, different dilutions of the anti-EF-Tu antibody (1∶50, 1∶100, or 1∶200) and rabbit pre-bleed serum were added to bacterial suspensions containing 5×10^7^ CFU/mL. The bacterial suspensions were then incubated for 1 h at 37°C at pH 5.0. The adhesion assay was performed as outlined above.

### Statistical Analyses

GraphPad Prism6 software was used for all statistical analyses. The statistical tests used to analyze each set of data are indicated in the figure legends. “n” represents the number of individual experiments.

## Supporting Information

Figure S1 **MS spectra of mucin oligosaccharides before and after chemical desialylation.** Desialylated mucin oligosaccharides were analyzed by MALDI-TOF/MS in negative ion mode (A) before or (B) after desialylation. Mass spectrum of (B) after desialylation; *m/z* 675, 878, 1040, 1243, 1389, 1852, 2055, and 2096 were not identified as desialylated mucin oligosaccharides (see Table S1).
(TIFF)

Figure S2 **Release of EF-Tu at different pH values.** Western blotting for detection of EF-Tu on the *L. reuteri* JCM1081 cell surface and in the supernatant obtained after cells had been incubated for 1 h at the indicated pH. For comparison, reactivity with anti-RNA polymerase antibodies is shown.
(TIFF)

Figure S3 **Adhesion of** ***L. reuteri*** **JCM1081 to mucin at different pH conditions.** Bacteria were pre-treated in DMEM at pH values ranging from 4.0 to 7.2. Adhesion assays were performed as described in the Materials and Methods. Asterisks indicate significant differences (*p<0.05) in adhesion, as analyzed by one-way ANOVA with *post hoc* Bonferroni test (n* = *4).
(TIFF)

Table S1 **The mass of sialylated and sulfated-mucin oligosaccharides calculated by MALDI-TOF/MS.**
(DOCX)
