# Correction: Identification and Characterization of Sulfated Carbohydrate-Binding Protein from *Lactobacillus reuteri*

**DOI:** 10.1371/journal.pone.0174257

**Published:** 2017-03-14

**Authors:** Keita Nishiyama, Ayaka Ochiai, Daigo Tsubokawa, Kazuhiko Ishihara, Yuji Yamamoto, Takao Mukai

There is an error in the caption for Fig 2, “Binding of His_6_-EF-Tu to sulfated glycolipids assessed by SPR analysis.” Please see the complete, correct Fig 2 caption here.

**Fig 2:**
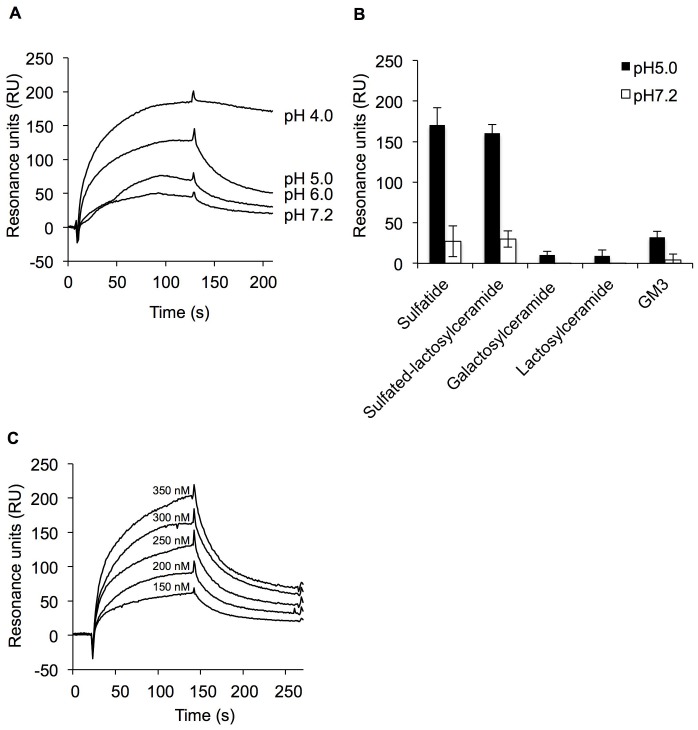
Binding of His_6_-EF-Tu to sulfated glycolipids assessed by SPR analysis. (A) Binding of His_6_-EF-Tu to sulfatide (SO_3_-3Galβ1Cer) at different pH values (pH 4.0, 5.0, 6.0, and 7.2). (B) Binding of His_6_-EF-Tu to various glycolipids: sulfatide, sulfated-lactosylceramide (SO_3_-3Galβ4Glcβ1Cer), galactosylceramide (Galβ1Cer), lactosylceramide (Galβ4Glcβ1Cer), and GM3 (NeuAcα3Galβ4Glcβ1Cer) at pH 5.0 and 7.2. Resonance units were measured at the start of dissociation. Error bars indicate standard deviations (n = 5). (C) Sensorgrams of the interaction of His_6_-EF-Tu with sulfatide at pH 5.0. Concentrations of sulfatide (from top to bottom) are as follows: 350, 300, 250, 200, and 150 nM. The K_D_ value is described in the text.
