# Supplementary figures and images for: Identification and Characterization of Sulfated Carbohydrate-Binding Protein from Lactobacillus reuteri
Source: PLoS One. 2013 Dec 31;8(12):e83703. doi: 10.1371/journal.pone.0083703 (PMC3877078; doi:10.1371/journal.pone.0083703)

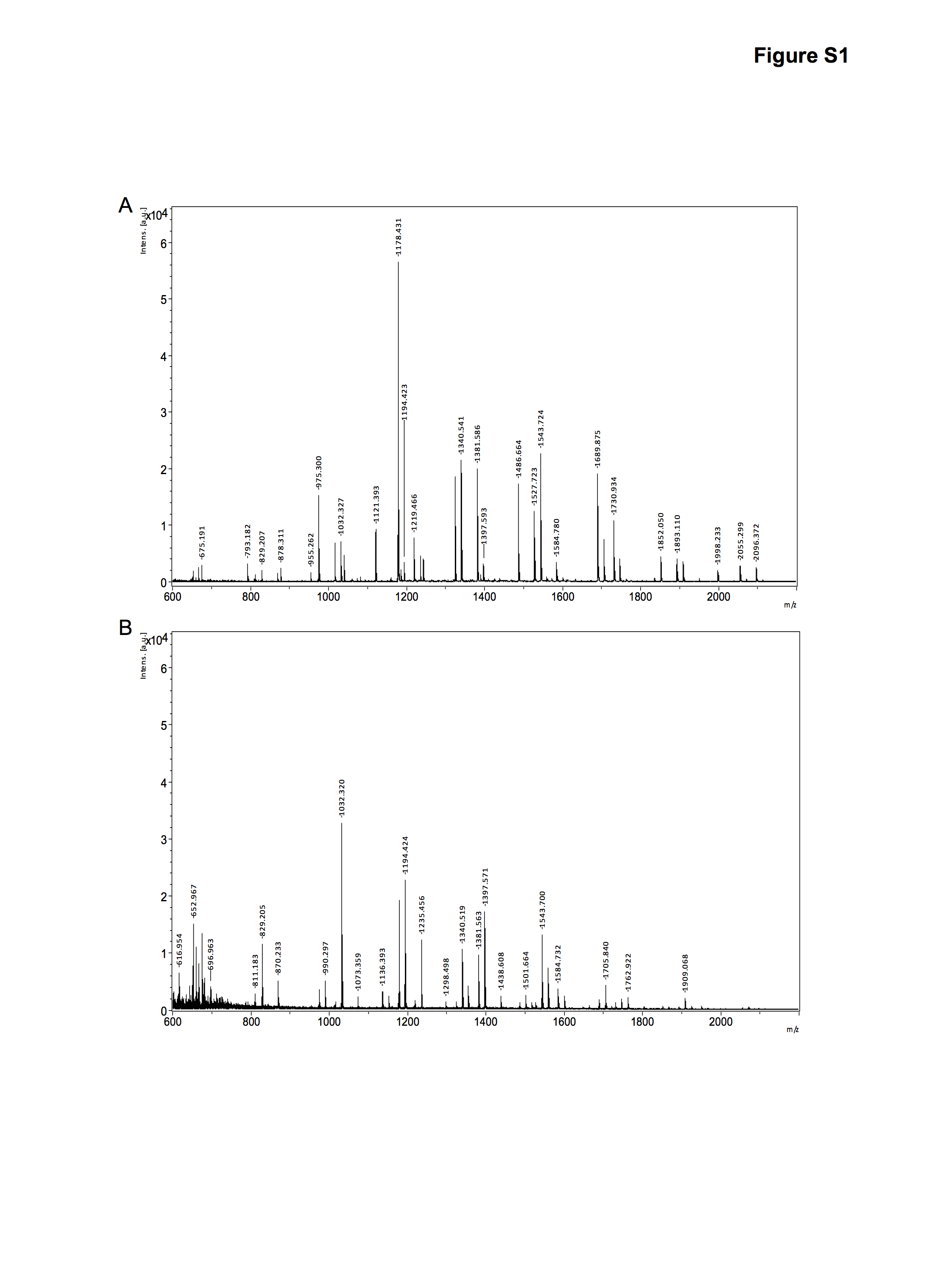

Supplement: Figure S1 — MS spectra of mucin oligosaccharides before and after chemical desialylation. Desialylated mucin oligosaccharides were analyzed by MALDI-TOF/MS in negative ion mode (A) before or (B) after desialylation. Mass spectrum of (B) after desialylation; m/z 675, 878, 1040, 1243, 1389, 1852, 2055, and 2096 were not identified as desialylated mucin oligosaccharides (see Table S1). (TIFF) [file pone.0083703.s001.tiff]

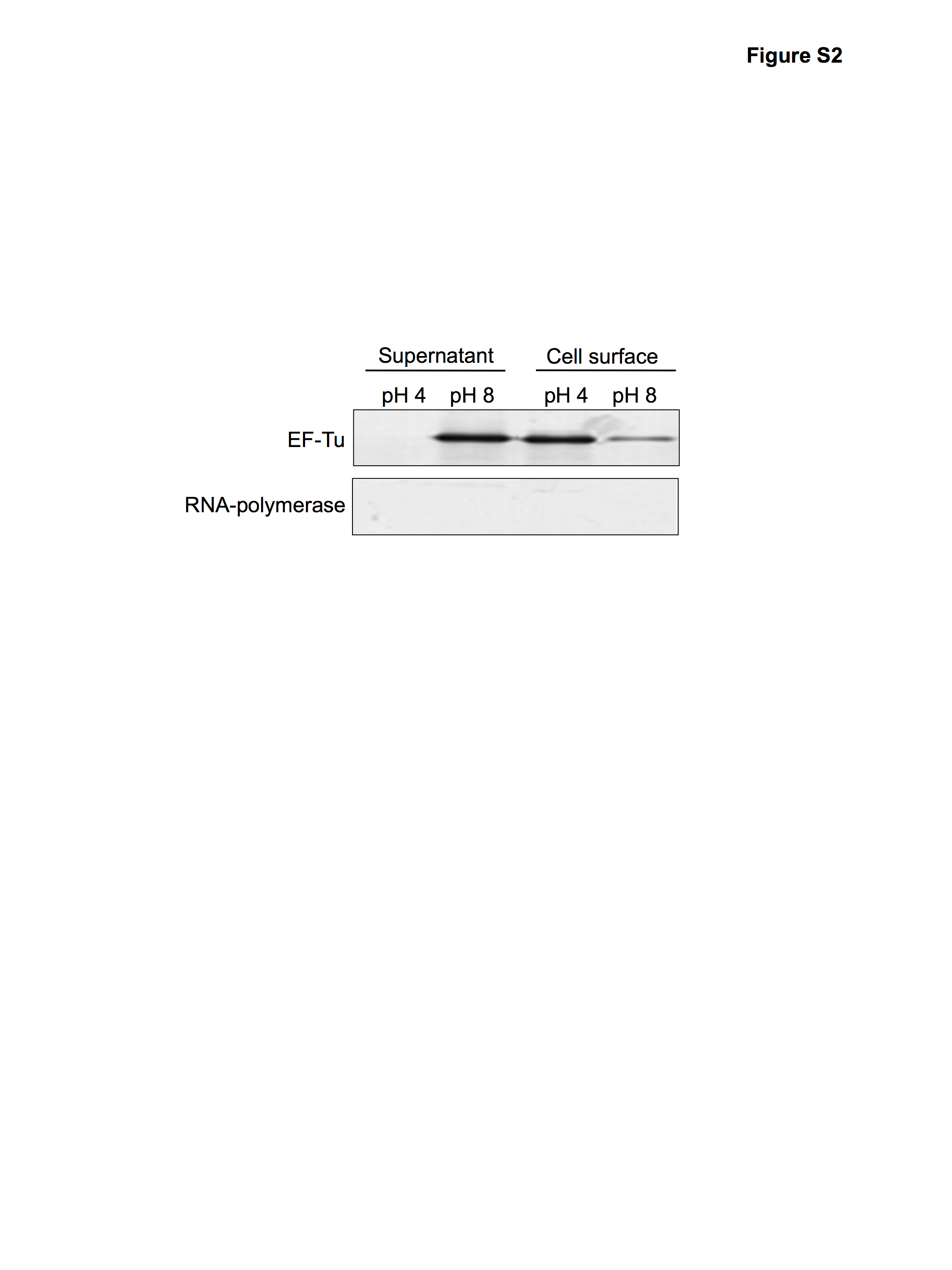

Supplement: Figure S2 — Release of EF-Tu at different pH values. Western blotting for detection of EF-Tu on the L. reuteri JCM1081 cell surface and in the supernatant obtained after cells had been incubated for 1 h at the indicated pH. For comparison, reactivity with anti-RNA polymerase antibodies is shown. (TIFF) [file pone.0083703.s002.tiff]

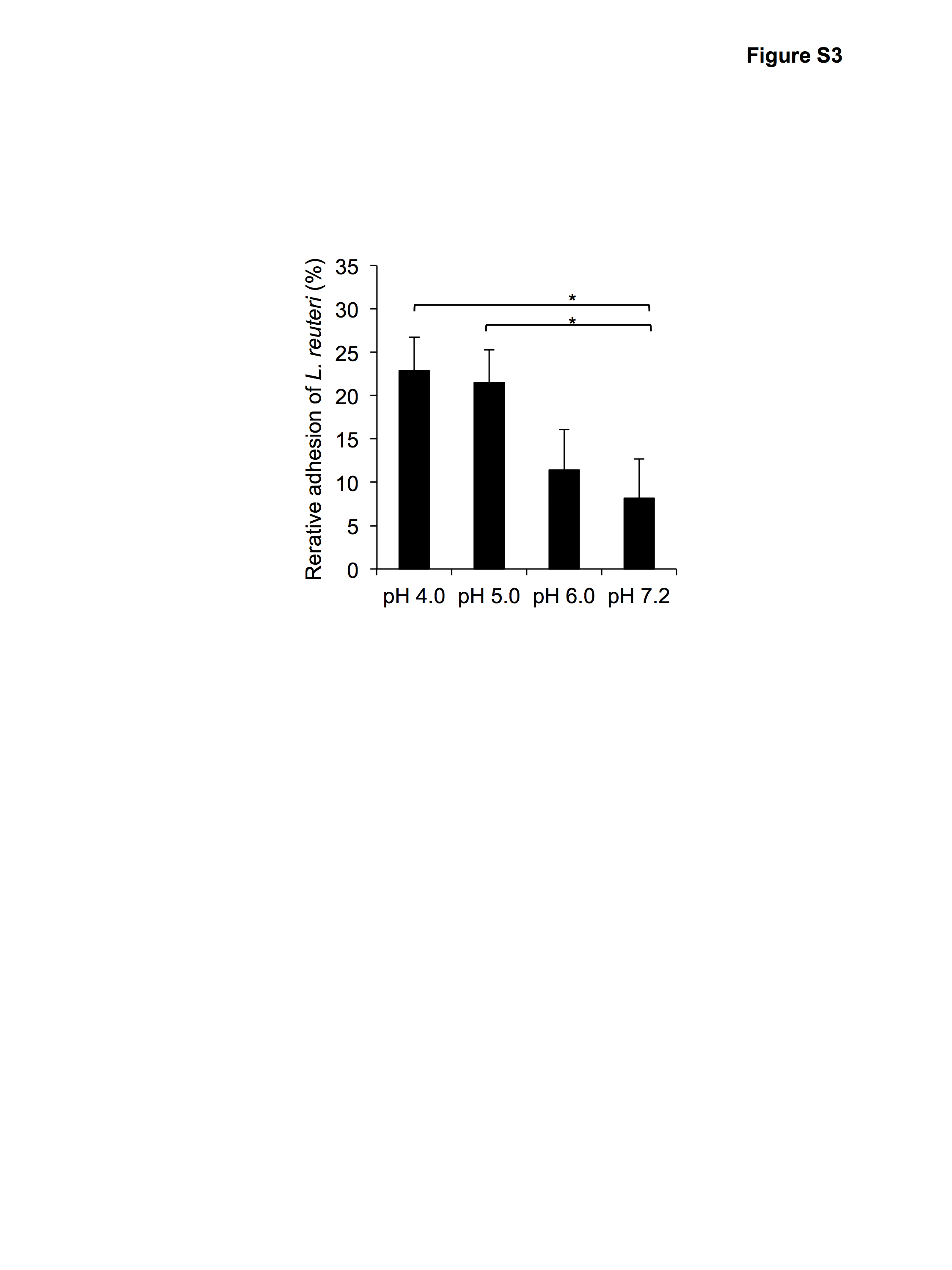

Supplement: Figure S3 — Adhesion of L. reuteri JCM1081 to mucin at different pH conditions. Bacteria were pre-treated in DMEM at pH values ranging from 4.0 to 7.2. Adhesion assays were performed as described in the Materials and Methods. Asterisks indicate significant differences (*p<0.05) in adhesion, as analyzed by one-way ANOVA with post hoc Bonferroni test (n = 4). (TIFF) [file pone.0083703.s003.tiff]
