# Supplementary material for: Identification and Characterization of Sulfated Carbohydrate-Binding Protein from Lactobacillus reuteri
Source: PLoS One. 2013 Dec 31;8(12):e83703. doi: 10.1371/journal.pone.0083703 (PMC3877078; doi:10.1371/journal.pone.0083703)
Supplement: Table S1 — The mass of sialylated and sulfated-mucin oligosaccharides calculated by MALDI-TOF/MS. (DOCX) [file pone.0083703.s004.docx]

**Table S1**

| [M-H]^-^ (m/z) | Expected composition of  sialyl-oligosaccharide-alditols | Non-treated | De-sialylaed |
| --- | --- | --- | --- |
| 675.24 | (NeuAc)(Hex)GalNAc-ol | + | − |
| 878.31 | (NeuAc)(Hex)(HexNAc)GalNAc-ol | + | − |
| 1040.4 | (NeuAc)(Hex)_2_(HexNAc)GalNAc-ol / (NeuGc)(dHex)(Hex)(HexNAc)GalNAc-ol | + | − |
| 1243.54 | (NeuAc)(Hex)_2_(HexNAc)_2_GalNAc-ol / (NeuGc)(dHex)(Hex)(HexNAc)_2_GalNAc-ol | + | − |
| 1389.59 | (NeuAc)(dHex)(Hex)_2_(HexNAc)_2_GalNAc-ol / (NeuGc)(dHex)_2_(Hex)(HexNAc)_2_GalNAc-ol | + | − |
| 1852.05 | (NeuAc)(Hex)_2_(HexNAc)_5_GalNAc-ol / (NeuGc)(dHex)(Hex)(HexNAc)_5_GalNAc-ol | + | − |
| 2055.3 | (NeuAc)(Hex)_2_(HexNAc)_6_GalNAc-ol / (NeuGc)(dHex)(Hex)(HexNAc)_6_GalNAc-ol | + | − |
| 2096.37 | (NeuAc)(Hex)(HexNAc)_7_GalNAc-ol | + | − |

| [M-H]^-^ (m/z) | Expected composition of  sulfated-oligosaccharide-alditols | Non-treated | De-sialylaed |
| --- | --- | --- | --- |
| 829.21 | (SO_3_H)(Hex)_3_(HexNAc)GalNAc-ol | + | + |
| 870.24 | (SO_3_H)(Hex)(HexNAc)_2_GalNAc-ol | + | + |
| 975.5 | (SO_3_H)(dHex)(Hex)_2_(HexNAc)GalNAc-ol | + | + |
| 1016.34 | (SO_3_H)(dHex)(Hex)(HexNAc)_2_GalNAc-ol | + | − |
| 1032.55 | (SO_3_H)(Hex)_2_(HexNAc)_2_GalNAc-ol | + | + |
| 1073.34 | (SO_3_H)(Hex)(HexNAc)_3_GalNAc-ol | − | + |
| 1121.58 | (SO_3_H)(dHex)_2_(Hex)_2_(HexNAc)GalNAc-ol | + | − |
| 1178.61 | (SO_3_H)(dHex)(Hex)_2_(HexNAc)_2_GalNAc-ol | + | + |
| 1194.42 | (SO_3_H)(Hex)_3_(HexNAc)_2_GalNAc-ol | + | + |
| 1219.47 | (SO_3_H)(dHex)(Hex)(HexNAc)_3_GalNAc-ol | + | − |
| 1235.46 | (SO_3_H)(Hex)_2_(HexNAc)_3_GalNAc-ol | + | + |
| 1324.54 | (SO_3_H)(dHex)_2_(Hex)_2_(HexNAc)_2_GalNAc-ol | + | + |
| 1340.54 | (SO_3_H)(dHex)(Hex)_3_(HexNAc)_2_GalNAc-ol | + | + |
| 1381.59 | (SO_3_H)(dHex)(Hex)_2_(HexNAc)_3_GalNAc-ol | + | + |
| 1397.59 | (SO_3_H)(Hex)_3_(HexNAc)_3_GalNAc-ol | + | + |
| 1486.66 | (SO_3_H)(dHex)_2_(Hex)_3_(HexNAc)_2_GalNAc-ol | + | − |
| 1527.72 | (SO_3_H)(dHex)_2_(Hex)_2_(HexNAc)_3_GalNAc-ol | + | − |
| 1543.72 | (SO_3_H)(dHex)(Hex)_3_(HexNAc)_3_GalNAc-ol | + | + |
| 1559.7 | (SO_3_H)(Hex)_4_(HexNAc)_3_GalNAc-ol | − | + |
| 1584.73 | (SO_3_H)(dHex)(Hex)_2_(HexNAc)_4_GalNAc-ol | + | + |
| 1600.76 | (SO_3_H)(Hex)_3_(HexNAc)_4_GalNAc-ol | − | + |
| 1689.88 | (SO_3_H)(dHex)_2_(Hex)_3_(HexNAc)_3_GalNAc-ol | + | − |
| 1705.84 | (SO_3_H)(dHex)(Hex)_4_(HexNAc)_3_GalNAc-ol | + | + |
| 1730.92 | (SO_3_H)(dHex)_2_(Hex)_2_(HexNAc)_4_GalNAc-ol | + | + |
| 1746.9 | (SO_3_H)(dHex)(Hex)_3_(HexNAc)_4_GalNAc-ol | + | + |
| 1762.92 | (SO_3_H)(Hex)_4_(HexNAc)_4_GalNAc-ol | − | + |
| 1909.07 | (SO_3_H)(dHex)(Hex)_4_(HexNAc)_4_GalNAc-ol | + | + |

dHex, deoxy-hexose; GalNAc, *N*-acetylgalactosamine; Hex, hexose; HexNAc, *N*-acetylhexosamine; NeuAc, *N*-acetylneuraminic acid; NeuGc, *N*-glycolylneuraminic acid; SO_3_H, sulfo group.
